# RIPK3-Dependent Necroptosis Is Induced and Restricts Viral Replication in Human Astrocytes Infected With Zika Virus

**DOI:** 10.3389/fcimb.2021.637710

**Published:** 2021-03-16

**Authors:** Chunxia Wen, Yufeng Yu, Chengfeng Gao, Xian Qi, Carol J. Cardona, Zheng Xing

**Affiliations:** ^1^ Medical School, Jiangsu Provincial Key Laboratory of Medicine, and the State Key Laboratory of Pharmaceutical Technology, Nanjing University, Nanjing, China; ^2^ Jiangsu Provincial Center for Disease Control and Prevention, Nanjing, China; ^3^ Department of Veterinary Biomedical Sciences, College of Veterinary Medicine, University of Minnesota at Twin Cities, Saint Paul, MN, United States

**Keywords:** necroptosis, astrocytes, RIPK1, RIPK3, Zika virus

## Abstract

Apoptosis, pyroptosis and necroptosis are regulated processes of cell death which can be crucial for viral disease outcomes in hosts because of their effects on viral pathogenicity and host resistance. Zika virus (ZIKV) is a mosquito-borne flavivirus, which infects humans and can cause neurological disorders. Neural developmental disorders and microcephaly could occur in infected fetuses. Several types of nervous cells have been reported to be susceptible to ZIKV infection. Human astrocytes play important roles in the nutritional support and defense of neurons. In this study, we show that human astrocytes are susceptible to ZIKV infection and undergo progressive cell death after infection. In infected astrocytes we detected no cleavage or activation of pro-caspase-3 and pro-caspase-1. Apoptotic substrates and increased secretion of interleukin (IL)-1β or IL-18 were not detected, either. These ruled out the occurrence of apoptosis or pyroptosis in ZIKV-infected astrocytes. We detected, however, an increase of phosphorylated receptor-interacting serine/threonine-protein kinase (RIPK)1, RIPK3, and mixed lineage kinase domain-like (MLKL) protein, indicating that programmed necrosis, or necroptosis, was induced in infected astrocytes. The phosphorylation and cell death were inhibited in cells pre-treated with GSK’872, an inhibitor of RIPK3, while inhibition of RIPK1 with an inhibitor, Necrostatin-1, had no effect, suggesting that ZIKV-induced necroptosis was RIPK1-independent in astrocytes. Consistent with this finding, the inhibition of RIPK1 had no effect on the phosphorylation of MLKL. We showed evidence that MLKL phosphorylation was RIPK3-dependent and ZBP-1, which could stimulate RIPK3, was upregulated in ZIKV-infected astrocytes. Finally, we demonstrated that in GSK’872-pre-treated astrocytes, viral replication increased significantly, which indicates that necroptosis may be protective against viral replication in astrocytes. Our finding that astrocytes uniquely underwent necroptosis in response to ZIKV infection provides insight and helps us better understand the viral pathogenesis in the ZIKV-infected central nervous system.

## Introduction

Zika virus (ZIKV) is a mosquito-borne flavivirus in the *Flaviviridae* family (Marchette et al., 1969; Sukupolvi-Petty et al., 2013; Diallo et al., 2014), first isolated from a sentinel rhesus macaque in Zika forest of Uganda in 1947 (Dick et al., 1952; Duffy et al., 2009). ZIKV has spread intercontinentally in the past decades and evolved in recent decades into African and Asian lineages (Wang et al., 2017). ZIKV has a single-stranded and positive-sense RNA genome encoding a long polyprotein, which is post-translationally cleaved and processed into envelope protein (E), capsid protein (C), and the precursor of membrane (prM) and seven nonstructural proteins (NS1-NS5) in infected cells (Fernandez-Garcia et al., 2009; Hamel et al., 2015; Shi and Gao, 2017). Infection with ZIKV was thought to be benign in humans, and the virus existed in obscurity for sixty years after it was first recognized in human patients in 1953 in Nigeria (Hayes, 2009; Brasil et al., 2016). That changed in 2013-2014 when an outbreak of ZIKV in French Polynesia was reported to have an association with neurological disease. In early 2016 the WHO announced a World Health Emergency due to massive ZIKV outbreak characterized by severe fetal microcephaly cases in South America (Bell et al., 2016; De Carvalho et al., 2016; Blish, 2017).

Cell death is one of host responses to viral infections. There are several types of cell death, which include necrosis, apoptosis, pyroptosis, and necroptosis (Chu and Ng, 2003; Kaczmarek et al., 2013; Nogusa et al., 2016). Apoptosis is programmed cell death dependent on a cascade of protease activation, is non-inflammatory and can have characteristic morphological features including cell shrinkage, nuclear condensation, and the plasma membrane blebbing (Perng et al., 2000; Rossman and Lamb, 2009). Many viruses can inhibit host apoptotic processes using various strategies likely to circumvent restrictions of viral replication in certain types of infections resulting from apoptosis. Pyroptosis is a process by which a molecular complex called inflammasome is assembled resulting in activation of pro-caspase-1 or pro-caspase-11 that consequently causes the cleavage of pro-IL-1β and pro-IL- 18 as well as gasdermin D (GSDMD). While mature IL-1β and IL-18 are released extracellularly to initiate proinflammatory responses, cleaved GSDMD aggregates to form oligomers, which are translocated to the plasma membrane to form pores causing cell death and the further release of proinflammatory factors that exacerbate inflammation in the site of infection. Inflammasomes are composed of pro-caspase-1, apoptosis-associated speck-like protein containing a caspase recruitment domain (ASC) and NOD2-like receptors (NLRs) (Man et al., 2017; Lee et al., 2018). On the other hand, necroptosis is a highly inflammatory cell death process, which is caspase-independent and initiated by necrosomes composed of RIPK1, RIPK3, and MLKL. Necroptosis is the form of programmed cell death orchestrated by RIPK1 and/or RIPK3 (Galluzzi et al., 2012; Weinlich et al., 2017) which activates executioner MLKL in the necrosomes (Vandenabeele et al., 2010; Kaczmarek et al., 2013). Upon stimulation MLKL is phosphorylated by RIPK3, forming oligomers which are translocated to and disrupt the plasma membrane causing cell swelling, rupture, and release of intracellular damage-associated molecular patterns (DAMPs), including IL-1α, HMGB-1, etc. Although the process was highly inflammatory, necroptosis can be a host defense against intracellular infection (Cho et al., 2011; Kaiser et al., 2013). *In vivo* studies showed that RIPK3^-/-^ mice were more susceptible to HSV-1 infection and had elevated virus loads (Huang et al., 2015).

Numerous studies have indicated that ZIKV can inhibit neurogenesis and induce apoptosis and autophagy in human fetal neural stem cells. ZIKV infection leads to complicated pathogenesis in which not only neurons but also glial cells are implicated. In this study we show that human astrocytes are susceptible to ZIKV and ZIKV infection led to necroptotic cell death after apoptosis and pyroptosis were ruled out. In response to ZIKV infection astrocytes released proinflammatory cytokines, such as IL-6, IL-8, and interferon-β (IFN-β). Our data showed that the induced necroptosis could be protective since ZIKV replication was inhibited in the infected cells pre-treated with an inhibitor to suppress the activation of RIPK3. Our finding in this study demonstrated that ZIKV infected glial cells in the central nervous system led to necroptotic cell death and restriction of viral replication. Necroptosis therefore could play an important role in viral pathogenesis of neural disorders caused by ZIKV.

## Materials and Methods

### Cells and Virus

The human astrocyte cell line (U251), African green monkey kidney epithelial cells (Vero) and human intestinal epithelial cells (HT-29) were purchased from the Cell Bank of the Chinese Academy of Sciences (Shanghai, China). BHK21 cells and ZIKV (SZ01) were obtained from Dr Shibo Jiang, Fudan University, Shanghai. The cells were cultured in Dulbecco Modified Eagles Medium (DMEM) with high glucose (Gibco), supplemented with 10% heat-inactivated Fetal bovine serum (FBS, Gibco) at 37°C in a humidified atmosphere with 5% CO_2_.

### Antibodies and Reagents

Rabbit anti–pro-caspase-3 (9555S), rabbit-anti-cleaved caspase3 (9664S), rabbit anti-pro-PARP (9532S), rabbit-anti-cleaved PARP (9541S), and rabbit-anti-phospho-RIP1(44590S) antibodies were purchased from Cell Signaling Technology (Beverly, MA). Rabbit anti-pro-caspase-1 (ab179515), rabbit anti-phospho-MLKL (ab187091) and rabbit anti-phospho RIPK3 (209384) antibodies were purchased from Abcam (Cambridge, MA). Rabbit anti-RIPK1antibody (A7414) was purchased from ABclonal (Wuhan, Hubei, China). Rabbit anti-RIPK3 (17563-I-AP), Mouse anti-MLKL (66675-I-Ig) t antibodies were purchased from Proteintech (Wuhan). Rabbit anti-Zika protein E antibody (B1845) was purchased from Biodragon-Immunotech (Beijing, China). Mouse anti-β-actin (BA2305) and GAPDH (A00227) antibody was purchased from BOSTER (Wuhan). Annexin V-FITC Apoptosis Detection Kit was purchased from KeyGEN Bio Tech (Nanjing, Jiangsu, China). ELISA kits for human IL-6, IL-8, IL-1β, IL-18, IFN-β and tumor necrosis factors were purchased from Multi Sciences (Hangzhou, Zhejiang, China). Human HMGB-1 ELISA Kit was purchased from Abclonal.

### Virus Infection and Titration

ZIKV strain SZ01 stock was propagated in Vero cells after inoculating the culture at a multiplicity of infection (MOI) of 0.01 and harvested at 120 h p.i. The virus stock was titrated by plaque forming assay on BHK21 cells. For the plaque forming assay, serially diluted virus from 10^-2^ to 10^-6^ in DMEM was inoculated to BHK21 cells cultured in 12-well plates for 2 hrs. After the removal of the viral inoculum, a mixed overlay of 500 µl DMEM with 4% FBS and 500µl low-melt agarose (2%) dissolved by PBS was added into each well. The cultures were incubated for 5 days in an incubator with 5% CO_2_ at 37°C before plaques were counted and infectious viral titers calculated for the virus stock.

### Cell Viability and Flow Cytometry

Cell viability was analyzed by 3-(4,5)-dimethylthiahiazo (-z-y1)-2,5-di- phenytetrazoliumromide (MTT) assay. Briefly, U251 cells were infected with ZIKV at a specific M.O.I. and the culture was incubated for variable times (from 12 to 72 h) before addition of MTT in the culture at 0.5 mg/ml. The culture was incubated for another 4 h before the cultural medium was taken for OD measurement. Survival rates of cells were expressed as the ratio of OD_570_ of the infected cell culture to OD_570_ of the uninfected or control cell culture. The assay was performed in triplicates for each sample.

Cell death was quantified in infected and control cells, which underwent necrosis or apoptosis using flow cytometric analysis. Infected or uninfected cells were collected at various time points. After washes twice with PBS, the cells were co-stained with annexin V and propidium iodide (PI) for 15 min on ice. After thorough washes, the cells were subjected to flow cytometry using a BD FACS flow cytometer. According to the manufacturer’s protocol, the annexin V+ population represents early phase apoptosis, and the PI+ population represents necrosis, while the double positive population represents late phase apoptosis or necrosis (Yuan et al., 2019).

### Western Blot Analysis

Proteins were analyzed with western blot analysis. For analyzing mitochondrial proteins, cells were lysed with pre-cooled RIPA lysis buffer for 15 min. After high-speed centrifugation (12,000 g, 5 min), the supernatant was harvested. Protein concentration in the clarified cell lysates was quantified by measurement with a bicinchoninic acid (BCA) protein assay kit (Pierce). The cell lysates were electrophoresed by SDS-PAGE before proteins were transferred to an Immunoblot PVDF membrane (Millipore) for incubation with respective primary antibodies. After overnight incubation, the membrane was washed with TBST before incubation with an HRP-conjugated secondary antibody for signal development. Images were captured using a FluoroChem FC2 Imaging System (TANON). The grayscale values of the protein bands in the immunoblots were measured with TANON and relative levels were normalized to the densities of GAPDH or β-actin in the same blot.

### ELISA

Cell culture media were harvested at different time points from infected or uninfected U251 cells for centrifugation at 1000 g for 15 min. The clarified medium was subjected to quantitative measurements of IL-6, IL-8, IL-18, IL-1β, IFN-β and TNF-α in 96-well plates using antibody sandwich ELISA kits following the manufacturer’s instructions. After the medium was incubated for 3 h followed by six 15–30 sec soakings in wash solution, a biotinylated antibody specific for a cytokine at a dilution as instructed was added to each well. The plates were further incubated for 45 min followed by six washes. HRP-conjugated streptavidin was added to each well for 45 min prior to colorimetric development. The reaction was stopped by addition of an acidic stop solution (0.2 M sulfuric acid) and absorbance values at 405 nm were read using a SpectraMax 340PC microplate reader (Molecular Devices). Serial dilutions of the standard controls were prepared and used to plot a standard curve of absorbance utilizing linear regression analysis.

### Quantitative Real Time PCR

Total RNA was extracted from each sample with Trizol reagent (Invitrogen) and quantified before the RNA was used for reverse transcription (RT) with a PrimeScript RT reagent kit (TaKaRa) following the manufacturer’s protocol. Real-time PCR was performed with 1 µl cDNA in 10 µl with SYBR Green master mix (Vazyme) according to the manufacturer’s instructions. Sequences of the primers used for PCR were listed in a supplemental document (**Table S1**). Relative gene expression levels were normalized to a GAPDH control.

### Immunofluorescence

Infected and control cells were fixed with paraformaldehyde (4%) diluted in PBS for 20 min at room temperature. After washes by PBS, the cells were permeabilized with 0.1% Triton X-100 diluted in PBS for 10 min, followed by washes with PBS, and then blocked with 5% BSA at room temperature for 2 h. Antibodies specific for ZIKV proteins were added at 1:100 dilution for incubation at 4°C overnight. After washes with PBST four times, the cells were further incubated with Alexa Fluor 488-conjugated goat anti-mouse or goat anti-rabbit antibody at a 1:200 dilution for 1 h at 37°C. The cells were washed prior to staining the nuclei with DAPI (1:1000) for 10 min. After three washes the cells were covered with one droplet of anti-fade reagent (Sigma-Aldrich) and observed under an Olympus confocal laser scanning microscope.

### Statistical Analysis

Unpaired Student’s t-test was used to evaluate the data. The data shown are the mean ± SEM of three independent experiments. P ≤ 0.05 was considered statistically significant.

## Results

### Human Astrocytes Were Susceptible to ZIKV Infection

**Figure 1:**
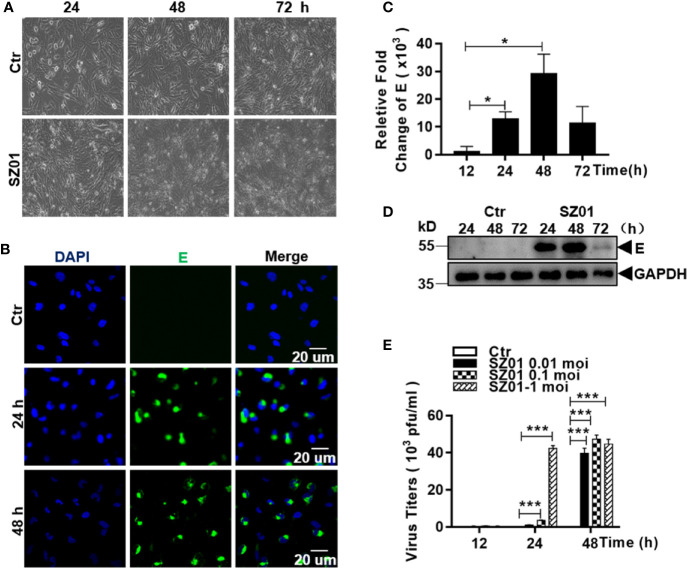
Susceptibility of human astrocytes to ZIKV and its pathologic effects on infected cells. **(A)** U251 cells were infected with ZIKV SZ01 at MOI of 1 and cytopathic effects (CPE) were observed at 24, 48, and 72 h p.i. (Magnification x40). **(B)** ZIKV E protein was expressed and detected by immunofluorescence assay with a specific antibody for E protein using confocal microscopy. **(C)** Replication of ZIKV genome in the infected cells. ZIKV E gene copies were quantified at various time points p.i. by real-time RT-PCR with specific primers for the viral E gene. Data were representative of three experiments and each experiment was performed in triplicate. Unpaired Student’s T-test was used to analyze the differences of data and error bars represent standard errors of the means. **(D)** Expressed viral E protein was detected in the infected cells by western blot with specific antibody (1:1000) for the E protein. **(E)** Viral titers in U251 cells infected with ZIKV at various MOI. Culture supernatants from the infected cells was collected at indicated time point p.i. and the virus titrated in BHK21 cells by a standard plaque assay (*p < 0.05; ***p < 0.001).

To investigate the susceptibility of human astrocytes to ZIKV, we infected human astrocytic glioma cell line U251 with ZIKV strain SZ01. The cells were inoculated at various doses (MOI of 0.01, 0.1 and 1). Morphology of the infected cells was observed and immunofluorescence staining (IFA) and quantitative real-time PCR were performed to detect viral antigen and quantify viral RNA in infected cells. As shown in **Figure 1A**, the morphology of the U251 cells, infected with SZ01 at an MOI of 1, became abnormal showing shrinkage with blurred boundaries. The cells eventually underwent lysis at later times post infection (p.i.) (**Figure 1A**). Viral envelope (E) protein could be detected at 24 and 48 h p.i. in the cytoplasm of infected cells by IFA (**Figure 1B**). Viral RNA for the E protein of ZIKV was measured by a real-time RT-PCR, which showed that viral RNA copy numbers of the E gene increased at 12 h and reached their peaks at 48 h p.i. (**Figure 1C**). Viral protein E could also be detected from the cell lysates prepared from the infected cells at various time points and the level reached its peak at 48 h p.i. as well (**Figure 1D**). To analyze replication of ZIKV in the astrocytes, culture medium of the SZ01-infetected cells was collected and titrated by a plaque-forming unit (PFU) assay for infectious viral titers. As shown in **Figure 1E**, ZIKA replicated robustly in the astrocytes with the infectious virus detected and the viral titers reached their peaks from 24 to 48 h p.i. with the initial MOI of 1 for inoculation. Collectively, the data demonstrated that human astrocyte U251 cells were susceptible to ZIKV, which replicated leading to cytopathic effects (CPE) in this type of glial cells.

### Cell Death Was Induced in Astrocytes in Response to ZIKV Infection

**Figure 2:**
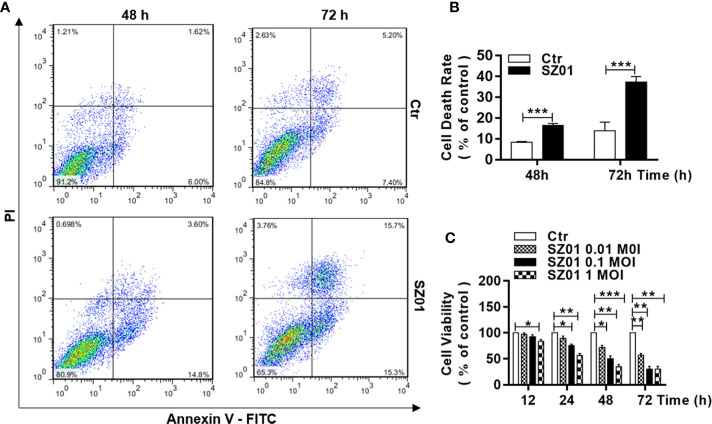
Cell death and reduced viability observed in human astrocytes infected with ZIKV. **(A)** U251 cells with or without ZIKV infection (MOI of 1) co-stained with Annexin V and PI at 48 and 72 h p.i. for flowcytometric analyses. **(B)** Quantitative analyses of cell death at various time points p.i. in U251 cells infected with ZIKV. ***p < 0.001. **(C)** Cell viability of U251 cells infected with ZIKV at various MOI was assessed with an MTT assay. Triplicate cultures were analyzed at the indicated time points (*p < 0.05; **p < 0.01; ***p < 0.001).

We quantified the cell death induced in U251 cells infected with ZIKV. The cells were infected with SZ01 ZIKV and harvested at 48 and 72 h p.i. for co-staining with annexin-V and propidium iodide (PI), and subsequently subjected to flow cytometry. As shown in **Figure 2A**, in comparison to those in non-infected cells, annexin V-FITC-positive and PI-positive cells increased significantly in infected cells at both 48 and 72 h p.i., which was quantified in **Figure 2B**. In general, cell viability deteriorated over time after inoculation at various doses. We infected the cells at three doses of MOI ranging from 0.1 to 1.0, the cells were harvested at 12, 24, 48, and 72 h p.i. for staining with a dye, 3-(4,5-dimethylthiazol-2-yl)-2,5- diphenylterazolium bromide (MTT), to assess cell viability. As shown in **Figure 2C**, there were significantly more cell death in ZIKV-infected cells compared to the non-infected cells throughout the time of infection and higher doses of the virus for infection caused more cell death.

### Apoptosis Was Not Induced in ZIKV-Infected Astrocytes

**Figure 3:**
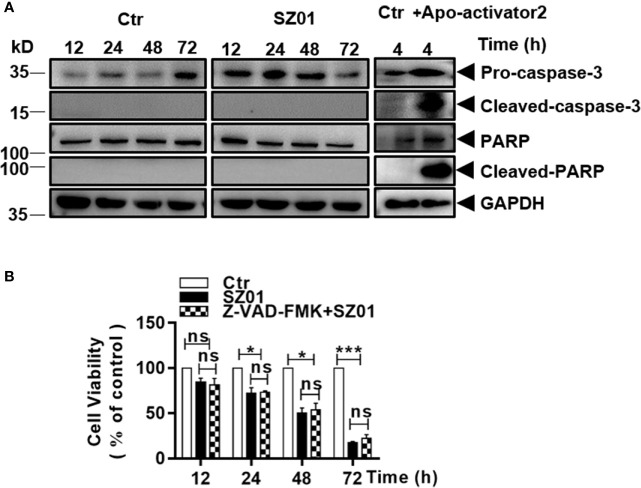
ZIKV did not induce apoptosis in human astrocytes. **(A)** U251 cells were infected with ZIKV (MOI of 1) and cell lysates prepared and subjected to western blot analyses with specific antibodies (1:1000) as indicated. HeLa cells were treated with or without Apoptosis activator2 (Apoa2, 10μM) and the lysates analyzed as a positive control for apoptosis. **(B)** Cell viability analyses in pre-treated U251 cells infected with ZIKV(MOI of 1). The cells were pre-treated with or without Z-VAD-FMK, a pan inhibitor of caspases, prior to ZIKV infection and analyzed for viability at various times p.i. with the MTT assay. The experiments were repeated at least three times (*p < 0.05; ***p < 0.001; ns, no significance).

We carried out additional studies to characterize the cell death induced in infected astrocytes. Cells were infected with ZIKV and cell lysates were prepared at various time points p.i. for western blot analysis. As shown in **Figure 3A**, cleaved caspase-3, the executioner caspase, was not detected in ZIKV-infected cells. Nor had we detected any cleaved form of PARP, a substrate of the executioner caspases in infected cells. As a control, HeLa cells were treated with apoptosis activator 2 (Apoa2; 10 µM) (Nguyen et al., 2010) for 4 hrs. Cell lysates were prepared and examined which showed presence of cleaved caspase-3 and cleaved PARP in response to Apoa2 (**Figure 3A**).

We also infected the cells, which were pre-treated with Z-VAD-FMK, a pan-caspase inhibitor, and the cells were harvested for flow cytometry after staining with MTT. In the cells pre-treated with Z-VAD-FMK, cell death increased at various time points p.i. but no significant differences were observed between the cell death in inhibitor treated and non-treated cultures (**Figure 3B**), suggesting that the cell death was caspase-independent and apoptosis was not activated or induced in ZIKV-infected astrocytes.

### ZIKV Infection Did Not Activate Inflammasome

**Figure 4:**
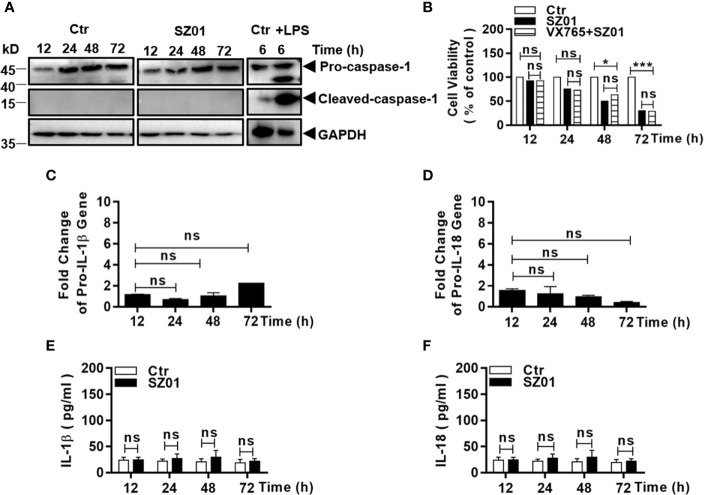
ZIKV did not activate pyroptosis in human astrocytes. **(A)** U251 cells were infected with 1 MOI of ZIKV and cell lysates were prepared at various time points p.i. and subjected to western blot analyses with specific antibodies (1:1000) as indicated. Monocytic THP-1 cells, were treated with or without lipopolysaccharide (LPS, 1μg/ml), and the lysates analyzed as a positive control for pyroptosis. **(B)** Cell viability was analyzed in pre-treated U251 cells infected with 1 MOI of ZIKV. The cells were pre-treated with or without VX765, an inhibitor of pro-caspase-1, prior to ZIKV infection and analyzed for viability at various times p.i. with the MTT assay. The experiments were repeated at least three times (ns p>0.05; *p < 0.05; ***p < 0.001). **(C, D)** No transcriptional changes occurred to pro-IL-1β and pro-IL-18 in infected cells. Total RNA was prepared from the U251 cells infected with ZIKV to measure mRNA transcript copies using quantitative real time PCR with specific primers for genes of IL-1β **(C)** and IL-18 **(D)**, respectively. **(E, F)** No change in secreted IL-1β and IL-18 levels in the cell cultures after ZIKV infection. Culture media was collected at various time points p.i. for measurement of IL-1β and IL-18 by ELISA. Each data point represents the mean values from triplicate cultures performed at least three times (ns p > 0.05).

We next tried to examine whether inflammasome was activated or pyroptosis was responsible for the cell death that occurred in the ZIKV-infected human astrocytes. Cells were infected with the virus and cell lysates were prepared at different time p.i. for western blot analysis. We found that pro-caspase-1 was not cleaved since active caspase-1 could not be detected (**Figure 4A**). As a control, THP-1 cells were treated with lipopolysaccharide (LPS, 1 µg/ml) for 6 h and the cell lysates were prepared and examined which showed the significantly increased cleaved caspase-1 (**Figure 4A**).

The astrocytes were also pre-treated with VX765, a caspase-1 inhibitor, prior to infection and the cell viability after infection was assessed with MTT staining. Likewise, the pre-treatment with VX765 had no impact on the increased death in ZIKV-infected cells (**Figure 4B**). Gene transcription or mRNA fold change of pro-IL-1β and pro-IL-18 did not increase as detected in total RNA from the infected cells in comparison with that from the non-infected cells by real-time PCR (**Figures 4C, D**).

In addition, the levels of secreted IL-1β and IL-18 in the culture media remained at the basal level which did not change through the course of infection (**Figures 4E, F**). Collectively, these results indicated that the cell death in ZIKV-infected U251 was not caused by activation of inflammasome and ZIKV infection did not induce pyroptosis in human astrocytes.

### ZIKV Infection Induced Necroptosis in Human Astrocytes

**Figure 5:**
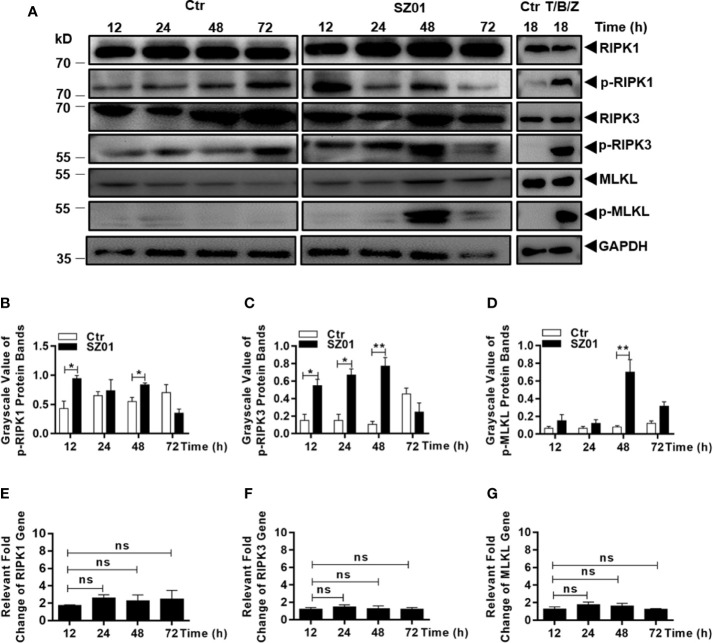
Necroptosis was induced by ZIKV-infected human astrocytes. **(A)** Increased phosphorylation of necroptosis-associated proteins was detected in ZIKV-infected cells. Cell lysates were prepared at various time points p.i. and subjected to SDS-PAGE and western blot analyses with specific antibodies (1:1000) for the proteins, either non-phosphorylated or phosphorylated, as indicated. The efficacies of the antibodies were confirmed with the lysates prepared from HT-29 cells treated with or without TNF-α (20 ng/ml), BV-6 (100 nM) and Z-VAD-FMK (20 µM) for induction of necroptosis. **(B–D)** Quantitative analyses of the gray scale values of the phosphorylated RIPK1 **(B)**, RIPK3 **(C)**, and MLKL **(D)** between ZIKV infected and non-infected cells at various time points p.i. **(E–G)**. Relative level of each protein was normalized to GAPDH at indicated time points. No changes of RIPK1, RIPK3 and MLKL at the transcriptional level in U251 cells after infection with ZIKV. Total RNA was prepared from infected cells at several time points p.i. for measuring mRNA transcript copy numbers by real time PCR with specific primers for RIPK1 **(E)**, RIPK3 **(F)**, and MLKL **(G)**, respectively. The experiments were repeated three times and the data are shown as means ± SEM (*p < 0.05; **p < 0.01; ns p > 0.05).

We finally examined the role of RIPKs in the cell death induced by ZIKV infection in human astrocytes. The cells were infected with ZIKV and lysed at different time points p.i. for western blot analyses with specific antibodies for RIPKs. As shown in **Figure 5A**, increased levels of phosphorylated RIPK1, RIPK3, and MLKL were detected between 12 and 48 h p.i. in ZIKV-infected astrocytes. Efficacies of the antibodies used in this study were confirmed in a human intestinal carcinoma cell line induced for necroptosis shown in the right panels, while the same stimuli did not induce cell death in U251 cells (data not shown). The differences in levels of the RIPK1, RIPK3, and MLKL phosphorylation, between the cells infected or uninfected with ZIKV, were statistically significant as quantitatively presented in **Figures 5B–D**, respectively. However, the gene transcription of RIPK1, RIPK3, and MLKL appeared not to be affected as shown in **Figures 5E–G**.

**Figure 6:**
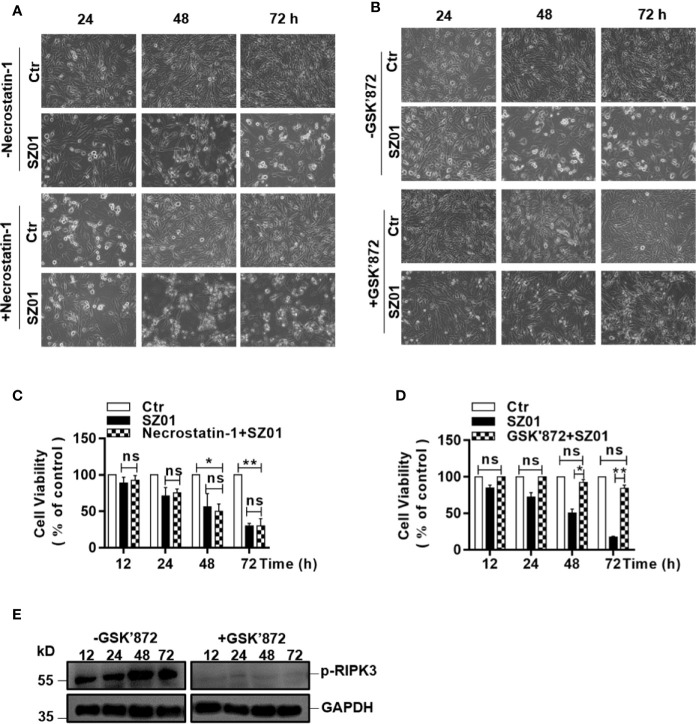
Effect of inhibiting the phosphorylation of RIPK1 or RIPK3 on necroptosis in ZIKV-infected human astrocytes. **(A, B)** U251 cells were pre-treated without (top) or with (bottom) necrostatin-1, an inhibitor of RIPK1 **(A)** or GSK’872, an inhibitor of RIPK3 **(B)**, prior to infection with ZIKV. Cell death was observed at 24, 48, and 72 h p.i. by light microscopy (magnification x 200). **(C, D)** Quantitative analyses of cell viabilities at 24, 48, and 72 h p.i. in U251 cells, untreated or pre-treated with necrostatin-1 **(C)** or GSK’872 **(D)**, prior to infection with ZIKV. The experiments were repeated three times and the values presented are means ± SEM (*p < 0.05; **p < 0.01; ns p > 0.05). **(E)** Inhibition of the RIPK3 phosphorylation. U251 cells were untreated or pre-treated with the inhibitor, followed by ZIKV infection. Cell lysates were prepared for SDS-PAGE and western blot analysis with indicated antibodies.

To confirm that necroptosis was induced, we next pre-treated the U251 cells with necrostatin-1, an inhibitor of RIPK1, followed by ZIKV infection. We monitored cell morphology and measured cell viability change at various time points p.i. As shown in **Figure 6A**, apparent cytopathic effects was shown in the pre-treated cells starting at 24 through 72 h p.i. compared to the infected cells without necrostatin-1 treatment, indicating that inhibition of RIPK1 was not sufficient to suppress the necroptosis induced in the infected astrocytes.

The fact that the cell death was not affected in the cells with only inhibited RIPK1 was confirmed quantitatively by cell viability assay with MTT staining performed on the U251 cells, pre-treated with or without necrostatin-1 (**Figure 6C**). There were no significant differences in loss of cell viability between the cells treated with or without necrostatin-1, the inhibitor of RIPK1.

We further pre-treated the cells with GSK’872, an inhibitor of RIPK3, followed by ZIKV infection, and observed the morphological integrity change of the cells p.i. As shown in **Figure 6B**, the ZIKA-infected cells pre-treated with GSK’872 remained unchanged in morphology in comparison to the uninfected cells p.i. In contrast, the infected cells without GSK’872 treatment underwent necrotic deterioration and the monolayer started to deteriorate after infection, indicating that the ZIKV-induced necroptosis was suppressed effectively when RIPK3 was inhibited in the cells (**Figure 6B**, bottom). The rescue of the cell death by the inhibition of RIPK3 by GSK’872 treatment was also confirmed quantitatively with a cell viability assay using MTT staining. Significant loss of cell viability was exhibited in the infected cells without RIPK3 inhibition while the GSK’872-pre-treated cells survived the ZIKV infection (**Figure 6D**). Inhibition of the RIPK3 phosphorylation by the GSK’872 was shown in the infected cells pre-treated with the compound in the western blot analysis (**Figure 6E**). Combined with the data shown earlier, ZIKV induced cell death was likely necroptotic, which could be RIPK1-independent. In another word, ZIKV induced necroptosis in human astrocytes could be mainly RIPK3-dependent.

### ZIKV Induced DAMPs and Proinflammatory Cytokines in U251 Cells

**Figure 7:**
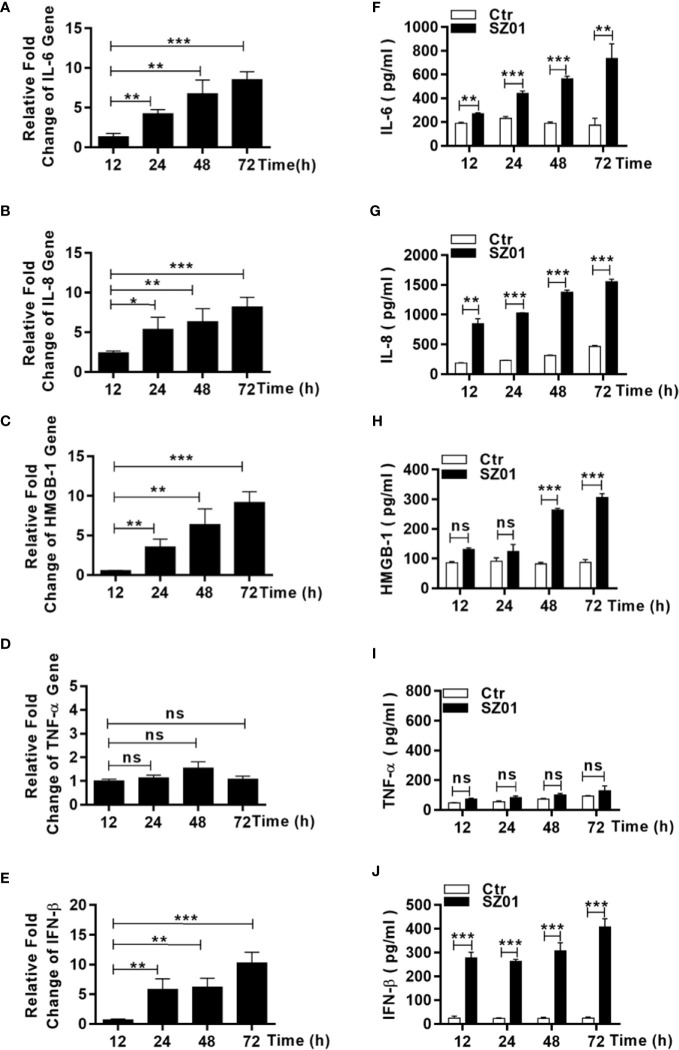
Upregulation and increased release of proinflammatory cytokines and DAMP from human astrocytes infected with ZIKV. **(A–E)** U251 cells were infected with ZIKV at an MOI of 1 and total RNA was prepared at various time points p.i. for measurement of mRNA transcript levels of selected cytokines and DAMP by real time RT-PCR with specific primers for IL-6 **(A)**, IL-8 **(B)**, HMGB-1 **(C)**, TNF-α **(D)**, and IFN-β **(E)**. **(F–I, J)** U251 cells were infected with ZIKV at an MOI of -1 and the culture media was collected at various time points p.i. for measurement of secreted protein levels of selected cytokines and DAMP by ELISA. IL-6 **(F)**, IL-8 **(G)**, HMGB-1 **(H)**, TNF-α **(I)**, and IFN-β **(J)** were tested with respective reagents. Values represent means ± SEM obtained from triplicate cultures and each test was repeated for at least three times (*p < 0.05; **p < 0.01; ***p < 0.001; ns p > 0.05).

To understand the mechanism how necroptosis was induced, we examined the induction of some proinflammatory cytokines and DAMP, which might be upregulated and/or released from ZIKA-infected astrocytes and subsequently have triggered astrocytotic necroptosis. The astrocytes were infected with ZIKV and total RNA were prepared from the infected cells at different points p.i. for real time RT-PCR. We aimed to measure copy numbers of selected DAMP and cytokine gene transcripts, including IL-6, IL-8, TNF-α, HMGB1, and IFN-β. As shown in **Figures 7A–C, E**, the levels of IL-6, IL-8, HMGB-1, and IFN-β RNA transcripts increased in a time-dependent manner after infection. Secreted IL-6, IL-8, HMGB-1, and IFN-β were also detected in the culture media of the infected cells collected at the various time points p.i. (**Figures 7F–H, J**). The gene transcript copies and secretion of TNF-α, however, remained unchanged at the basal level in the infected cells (**Figures 7D, I**). These data suggested that the increased proinflammatory cytokines, IL-6 and IL-8, as well as HMGB-1 could be critical to ZIKV-induced pathogenicity, contributed by astrocytes in the central nervous system.

As an inflammatory cytokine, TNF-α could induce signaling which leads to phosphorylation and activation of RIPK3. However, it was not significantly upregulated in ZIKV-infected astrocytes (**Figures 7E, F**). TNF-α probably was not the cause for initiating necroptosis in ZIKV-infected human astrocytes or may not induce necroptosis in human astrocytes since we did have observed that U251 remained healthy while treated with TNF-α along with inhibitors of pro-caspases (BV-6 and Z-VAD-FMK)(data not shown).

### Upregulation of Innate Sensor Expressions in Human Astrocytes Infected With ZIKV

**Figure 8:**
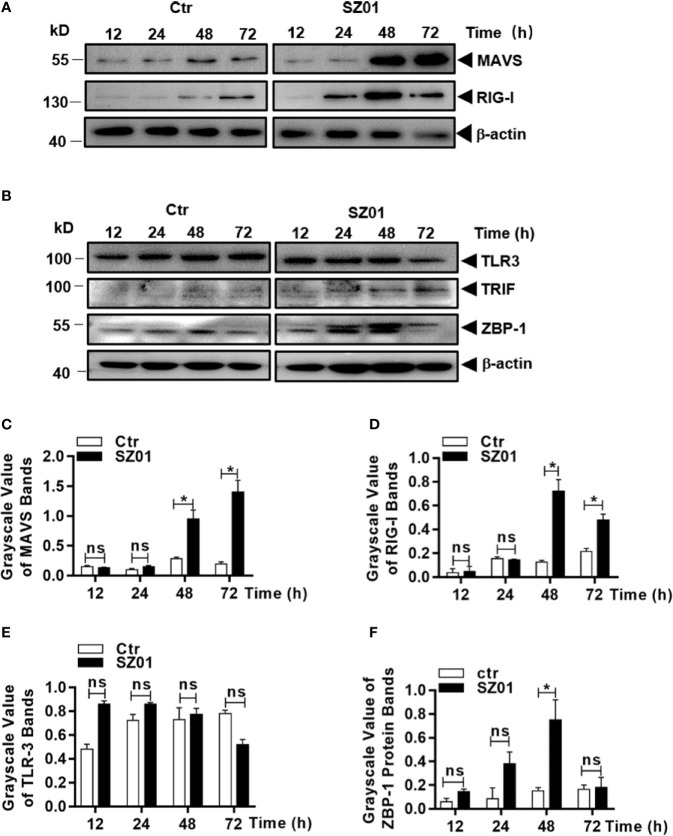
Upregulation of cellular sensors of viral nucleic acids in ZIKV-infected human astrocytes. U251 cells were infected with 1 MOI of ZIKV and cell lysates were prepared for SDS-PAGE analyses with specific antibodies (1:1000) as indicated for MAVS, RIG-1 **(A)**, or TLR3, TRIF, and ZBP-1 **(B)**. Grayscale values were analyzed for quantitative comparison of the protein expressions of MAVS **(C)**, RIG-1 **(D)**, TLR3 **(E)**, and ZBP-1 **(F)** in ZIKV-infected cells. Relative grayscale of each protein was normalized to β-actin at indicated time point. The experiments were repeated at least three times and the values presented were means ± SEM (*p < 0.05; ns p > 0.05).

RIPK3 can be activated in multiple mechanisms. Considering that RIPK3 can be phosphorylated and activated independent of RIPK1 and TNF-α in ZIKV-infected astrocytes, we examined whether innate sensors are associated with RIPK3 activation and necroptotic induction in human astrocytes. We analyzed the cell lysates prepared from ZIKV-infected U251 cells at various times points for detection of innate sensors responsible for signaling to viral infection. As shown in **Figures 8A**, the expression of MAVS and RIG-I were upregulated, together with induced expression of MDA5, which could lead to induction of type I IFN and cytokines as shown in **Figure 7**. IFN-inducible Z-DNA binding protein 1 (ZBP-1) was found to be upregulated as well (**Figure 8B**). The induction of MAVS, RIG-I, TLR-3 and ZBP-1 was quantitatively analyzed and significant increases of MAVS, RIG-I and ZBP-1 expressions were confirmed in ZIKV-infected astrocytes while TLR-3 remained unchanged as shown in **Figures 8C–F**.

### ZIKV-Induced Necroptosis Protected Against Viral Replication in Human Astrocytes

**Figure 9:**
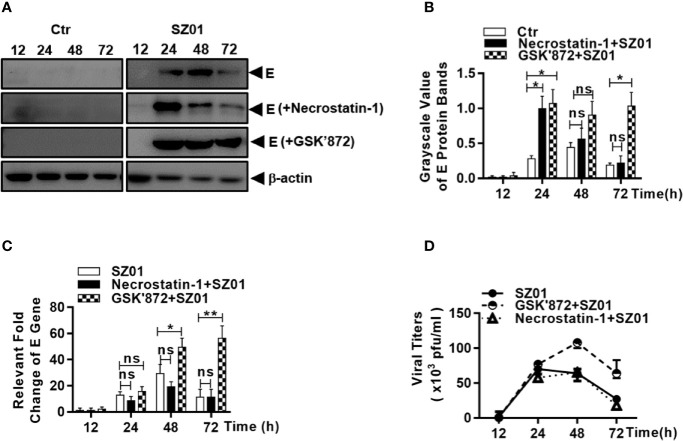
Necroptosis restricted viral replication in human astrocytes infected with ZIKV. U251 cells were untreated (left) or pre-treated with (right) necrostatin-1 or GSK’872 prior to infection with ZIKV. **(A)** Cell lysates were prepared from the infected cells at 12, 24, 48, and 72 h p.i. for SDS-PAGE and western blot analyses with anti-ZIKV E protein for levels of the viral protein E. **(B)** Quantitative analyses of the viral E protein levels in ZIKV-infected U251 cells pre-treated without or with necrostatin-1 or GSK’872. The E protein grayscale was normalized by β-actin at indicated time point. **(C)** Total RNA were prepared from the infected cells at 12, 24, 48, and 72 h p.i. for real time RT-PCR with primers specific for the viral E gene to measure the E RNA copy numbers in ZIKV-infected cells pre-treated with or without inhibitors. **(D)** Culture media was collected at various time points p.i. from ZIKV-infected cells, untreated or pre-treated with the inhibitors, for titration in Vero cells to determine infectious viral titers. The experiments were repeated for at least three times and the values presented were means ± SEM (*p < 0.05; **p < 0.01; ns p > 0.05).

We examined the effect of ZIKV-induced necroptosis on viral replication in human astrocytes. The U251 cells were pre-treated with either necrostatin-1 or GSK’872, followed by ZIKV infection. We analyzed the lysates collected from cells pre-treated with either necrostain-1 or GSK’872 and found that the ZIKV E protein expression was much higher in GSK’872-treated cells than in those treated with necrostatin-1 or those that were untreated (**Figures 9A**). The differences were quantitatively analyzed and exhibited in **Figure 9B**. Inhibition of viral replication was also confirmed in GSK’872-treated cells by measuring viral E gene copy numbers by real-time RT-PCR with total RNA prepared after the infection. As shown in **Figure 9C**, E gene copies increased significantly in GSK’872-treated cells compare to the untreated and necrostatin-1 treated cultures.

Finally, culture media was harvested at various time points p.i. and infectious viral titers were determined. As shown in **Figure 9D**, infectious viral titers were detected after 24 h p.i. and no differences in titers were detected between untreated cells and those pre-treated with necrostatin-1, the inhibitor of RIPK1, suggesting that a functional RIPK1 has no impact on viral replication. In contrast, viral titers were significantly higher in GSK’872-treated cells compared to untreated cells, further demonstrating that RIPK3-denpendent necroptosis induced by ZIKV infection can be protective against viral replication in human astrocytes.

## Discussion

ZIKV is a neurotropic flavivirus that can cause severe neurological disorders including microcephaly and cortical thinning during early development when the brain is infected by the virus passed to the fetus through the blood-placenta barrier (BPB). The brain can also be infected by the virus at later stages, including during early neonatal development. How various types of neural cells differ in their susceptibility to the virus and roles in contributing to neurological pathology remains a question. Recent studies have shown in a mouse model that astrocytes, distributed isolated throughout the brain, were the first cells targeted by the virus when the mouse was inoculated peripherally at birth (van den Pol et al., 2017). Release of ZIKV viruses from infected astrocytes would affect neurons and astrocytes could play a potentially significant role in initiating the brain infection and be critical in the development of ZIKV neural disorders.

Glial cells, of which astrocytes are one type, make up a large proportion of the nervous system. Once infected by viruses, glial cells can react by participating in immune responses in the brain. When infected by micro-organisms, astrocytes can produce many cytokines and proinflammatory DAMPs could be released which subsequently regulate innate and specific immunity in the brain. However, what exact roles astrocytes can play in ZIKV-infected brain remain largely uncharacterized. In this study we report that human astrocytes were susceptible to ZIKV and that infection led to cell death. While we were interested in the cytokines and DAMPs induced in ZIKV-infected astrocytes, which would contribute to the induction of innate or acquired immunity, we also examined the mechanism of death for ZIKV-infected astrocytes. We present evidence that the glial cells did not die of either apoptosis or pyroptosis. Instead ZIKV induced a necroptotic process in infected astrocytes, which could be suppressed by an inhibitor of RIPK3 to inhibit the formation of necrosome.

To respond to viral infection, host innate responses are activated which include induction of innate immunity with interferons and antiviral ISGs to defend against viral infection. As a part of innate responses, programed cell death can be triggered and may benefit the host or aggravate disease in the host. Apoptosis was induced in neurons when the brain was infected with ZIKV (Ho et al., 2017). In a fetal mouse model infected with ZIKV, the cell cycle was blocked in infected progenitor cells leading to differential deficiency and apoptosis of neurons (Li et al., 2016). Apoptosis has been reported in neural progenitor cells infected with ZIKV (Souza et al., 2016). In neonatal C57BL/6 mice infected with ZIKV, cleaved caspase-3 was detected in the brain (Huang et al., 2016). Glial cells may have fates different from neurons when infected with ZIKV. In the presence of extensive cell death after infection with ZIKV, we were unable to detect the activation of pro-caspase-8 and cleaved caspase-3, and neither did we detect substrates processed by active caspases indicating that apoptosis was not initiated in ZIKV-infected astrocytes.

Previous studies have shown that the secretion of proinflammatory cytokines such as IL-1β was induced through activation of the NLRP3 inflammasome, which was responsible for inflammatory responses (Wang et al., 2018). Presence of NLRP1, NLRP3, and AIM2 together with elevated IL-1β, IL-18, and IL-33 could be detected in the brain of ZIKV-infected patients with microcephaly (de Sousa et al., 2018a; de Sousa et al., 2018b). However, pyroptosis may not occur in astrocytes as shown in our study that no activation of pro-caspase-1 occurred and neither did secretion of IL-1β and IL-18 in ZIKV-infected astrocytes.

Necroptosis was identified in various tissues infected with viruses. MLKL was upregulated and phosphorylated in neurons of the mice infected with Japanese Encephalitis Virus (JEV) (Bian et al., 2017). Interaction of viral ICP6 and RIPK1 and RIPK3 led to induction of necroptosis in Herpes Simplex Virus-I (HSV-1)-infectedmurine fibroblasts, which suppressed viral replication (Huang et al., 2015). In our study increased phosphorylation of RIPK1, RIPK3, and MKLK in ZIKV-infected astrocytes was detected early in the infection but only RIPK3 and MLKL phosphorylation remained throughout the infection and cell death. Using an inhibitor of RIPK1 to pre-treat cells, necroptosis was not affected after viral infection indicating that the ZIKV-induced necroptosis was not RIPK1 dependent and neither RIPK3 nor necrosome was probably not activated by RIPK1 in astrocytes. The canonical necroptosis pathway could be bypassed by intracellular bacteria. In macrophages infected with Mycobacterium tuberculosis (Mtb) activation of RIPK1 was not required for necroptosis (Pajuelo et al., 2018). Activation of RIPK3 and MLKL, stimulated by the Mtb necrotizing toxin, would suffice for causing necroptosis in Mtb-infected macrophages.

Necroptosis can be triggered in the course of various viral infections. Viruses from many families are capable of inducing necroptosis, including cytomegalovirus (Upton et al., 2019), HSV-1 (Huang et al., 2015), vaccinia virus (Cho et al., 2009), and influenza A virus (Nogusa et al., 2016). RIPK3 plays a key role in activation of necrosome and necroptosis. Necroptosis may play a key role in pathogenesis in addition to its impact on viral replication in viral diseases. Knockout mice deficient of RIPK3 were highly susceptible to infection with HSV-1 (Cho et al., 2009) and poxvirus (Wang et al., 2014). In mice infected with MCMV, virus-encoded proteins inhibited RIPK3 and necroptosis, which promoted viral persistent infection (Upton et al., 2019).

Multiple mechanisms have been proposed for activating RIPK3-dependent necroptosis. In addition to the RIPK1, adaptor protein TRIF, which was essential for the induction of IFN in response to stimulation by TLR3 or TLR4, was capable of RIPK3 activation (Green, 2019). The interferon-inducible protein Z-DNA binding protein 1 (ZBP1) is an innate nucleotide sensor in the cytosol which could be sensed by influenza A virus to trigger the NLRP3 inflammasome and pyroptosis in murine macrophages (Kuriakose et al., 2016). ZBP1 can also activate RIPK3 in neurons infected with ZIKV (Daniels et al., 2019). However, instead of inducing a necroptosis in infected neurons, RIPK3 was activated by ZBP1 to further trans-activate upregulation of immunoresponsive gene 1 (IRG1), an metabolic enzyme, which exhibited antiviral ZIKV activity and inhibited ZIKV replication through production of itaconate by catalyzing cis-aconitate in mitochondria in neurons. In our study we observed upregulation of ZBP1 in ZIKV-infected astrocytes, which may help activate RIPK3. Interestingly necroptosis was induced upon RIPK3 and this cell death appeared to be RIPK3-dependent because it could be suppressed by an inhibitor of RIPK3, indicating that astrocytes may differ from neurons in RIPK3 signaling after cellular sensing of ZIKV infection. We have not examined the regulation of IRG1 transcription and itaconate production in the cytoplasm upon RIPK3 activation. However, it could not be ruled out that in astrocytes ZIKV-induced activation of RIPK3 might activate upregulation of IRG1 as well, which could independently contribute to the suppression of ZIKV replication. Our data suggest that RIPK3-dependent necroptosis was inhibitory to ZIKV replication, indicating that this cell death could be beneficiary to the host even though infected astrocytes died of the necroptotic signaling during the infection. Thus activation of RIPK3 can suppress ZIKV infection through various mechanisms depending on cell types in the brain that are infected.

Is necroptosis a stochastic choice for astrocytes infected with ZIKV? In influenza A virus-infected mouse embryonic fibroblasts or airway epithelial cells, RIPK3 was activated by ZBP1, which subsequently initiated parallel signaling of both necroptosis and apoptosis (Kuriakose et al., 2016). Both apoptosis and necroptosis proved to be stand-alone cell death mechanisms that restricted viral replication and contributed to host defense (Shubina et al., 2020; Zhang et al., 2020). The activation of RIPK3, which could be triggered by ZBP1, did not drive these parallel pathways in ZIKV-infected astrocytes as shown in our study in that no apoptotic signaling was detected. We may wonder why apoptosis was not a chosen path for cell death in astrocytes because viral spread could be restricted without an inflammatory response in the brain. Instead necroptosis is inflammatory resulting in the necrotic release of cytokines and DAMPs. A logic explanation would be that the apoptotic apparatus was shut down either due to a mechanistic deficiency or blockage by viral protein(s), so that only necroptosis was possible. Even though ZIKV can infect various cell types including neural progenitors and neurons in the brain, astrocytes may be the initial target of ZIKV throughout the brain. Elimination of the virus at the earliest stage would be of utmost importance in controlling the infection and preventing viral spread to other neural cell types with more benefits over risks. Elucidation of viral infection and mechanism of cell death in astrocytes shed lights on our further understanding of viral pathogenesis of ZIKV infection.

## Data Availability Statement

The original contributions presented in the study are included in the article/**Supplementary Material**. Further inquiries can be directed to the corresponding authors.

## Author Contributions

CW and ZX conceived and coordinated the study. CW, YY, and CG designed, performed, and analyzed the experiments shown in **Figures 1** through **9**. XQ provided reagents, technical assistance, and contributed to completion of the studies. CW, CC, and ZX wrote the paper. All authors contributed to the article and approved the submitted version.

## Conflict of Interest

The authors declare that the research was conducted in the absence of any commercial or financial relationships that could be construed as a potential conflict of interest.
