# Supplementary material for: RIPK3-Dependent Necroptosis Is Induced and Restricts Viral Replication in Human Astrocytes Infected With Zika Virus
Source: Front Cell Infect Microbiol. 2021 Mar 16;11:637710. doi: 10.3389/fcimb.2021.637710 (PMC8007970; doi:10.3389/fcimb.2021.637710)
Supplement: Supplementary file 1 [file Table_1.docx]

| Primer Name |  | Sequences（5’­3’） |
| --- | --- | --- |
| ZIKV-E | Forward | GCCACTTGAAATGTCGCCTGAA |
|  | Reverse | AAGTCCGCTGTAAAGTTCACCG |
| IL-1β | Forward | CCAGCTACGAATCTCCGACC |
|  | Reverse | TCGTGCACATAAGCCTCGTT |
| IFN-β | Forward | GCTTGGATTCCTACAAAGAAGCA |
|  | Reverse | ATAGATGGTCAATGCGGCGTC |
| IL-8 | Forward | ACATACTCCAAACCTTTCCACC |
|  | Reverse | AAAACTTCTCCACAACCCTCTG |
| IL-6 | Forward | GCCACTCACCTCTTCAGAACG |
|  | Reverse | CAGTGCCTCTTTGCTGCTTTC |
| IL-18 | Forward | TCTTCATTGACCAAGGAAATCGG |
|  | Reverse | TCCGGGGTGCATTATCTCTAC |
| HMGB1 | Forward | TGTCGGGAGGAGCATAAGAAG |
|  | Reverse | GGGCGATACTCAGAGCAGAAG |
| TNF-α | Forward | CCTCTCTCTAATCAGCCCTCTG |
|  | Reverse | GAGGACCTGGGAGTAGATGAG |
| RIPK1 | Forward | GACTATGAGCGAGATGGACTGAA |
|  | Reverse | AAGGTCGATCCTGGAACACTG |
| RIPK3 | Forward | GGCGGTCAAGATCGTAAACTC |
|  | Reverse | TCTGGTCGTGCAGGTAAAACA |
| MLKL | Forward | TGCCCTGGAGGAGGCTAATG |
|  | Reverse | CAACCTGAAGTAACAGCGAGA |
| GAPDH | Forward | TGCACCACCAACTGCTTAGC |
|  | Reverse | GGCATGGACTGTGGTCATGAG |

Table S1 Sequences of the Primers Using in RT-PCR
